# Perioperative therapies for metastatic renal cell carcinoma

**DOI:** 10.20517/2394-4722.2025.43

**Published:** 2025-08-03

**Authors:** Matthew P. Lierz, Allen Enrique D. Siapno, Adam Khorasanchi, Shawn Dason, Yuanquan Yang, Eric A. Singer

**Affiliations:** 1 Division of Urologic Oncology, The Ohio State University Comprehensive Cancer Center, Columbus, OH 43221, USA; 2 Division of Medical Oncology, The Ohio State University Comprehensive Cancer Center, Columbus, OH 43221, USA

**Keywords:** Metastatic renal cell carcinoma, perioperative therapy, neoadjuvant, adjuvant, cytoreductive nephrectomy, immune checkpoint inhibitor, tyrosine kinase inhibitor, clinical trials, biomarkers

## Abstract

Systemic therapy for metastatic renal cell carcinoma (mRCC) has advanced considerably over the past decade. Studies of systemic therapies in the adjuvant, neoadjuvant, and now perioperative settings as solitary, dual, and triplet combinations are underway. The timing of systemic therapy relative to extirpative surgery has yielded varying results. With the rise of novel therapeutics, new methods for risk stratification and patient selection, including initial biomarker evaluation, have begun. This perspective aims to summarize investigations into perioperative mRCC therapies and discuss the foundations of these concepts based on recent adjuvant and neoadjuvant trials, and to discuss future directions in this space.

## INTRODUCTION

Renal cell carcinoma (RCC), the 7th most incident cancer in the United States in 2024, presents with a 16% rate of regional lymph node involvement and a 15% rate of metastatic disease (mRCC) at diagnosis^[1]^. The 2005 US Food and Drug Administration (FDA) approval of sunitinib [anti-VEGF tyrosine kinase inhibitor (TKI)] revolutionized targeted therapy for mRCC. The introduction of immune checkpoint inhibitors (ICIs) targeting the PD-1/PD-L1 pathway transformed treatment approaches, described as “seismic” in the 2022 American Society of Clinical Oncology (ASCO) clear cell renal cell carcinoma (ccRCC) guidelines, with “substantial efficacy” cited in other international guidelines^[2,3]^. Notably, metastatic RCC patients with PD-L1-expressing tumors have been shown to exhibit higher response rates and improved outcomes following ICI treatment^[4]^. Similarly, ICIs have been characterized by high efficacy rates in patients with urothelial carcinoma (UC), and are recommended for the frontline management of patients with advanced disease^[5,6]^.

Dual ICI and TKI/ICI combinations are first-line systemic treatment for mRCC. Research reviewing the role and timing of surgery, and integration of targeted therapies is ongoing and critical to optimal patient and therapy selection^[5,6]^. This perspective examines current mRCC treatment, perioperative therapy, and future directions, highlighting the fervor within this space and the need for continued support to conduct more robust clinical trials.

## UPFRONT CYTOREDUCTIVE NEPHRECTOMY

CN for mRCC aims to reduce symptoms, treat paraneoplastic syndromes, and improve progression-free survival (PFS) and OS. Deferred CN (dCN) after neoadjuvant therapy targets surgical complete remission and enables patient selection by excluding those with rapid progression. ASCO’s 2022 mccRCC guideline designates patients with synchronous mRCC with the majority of the tumor burden in the kidney, good ECOG status, and no brain, bone, or liver metastases as “optimal” candidates for CN^[2,7]^.

The SURTIME and CARMENA trials, although completed before first-line ICI therapy development, assessed the combination of CN and TKI in the treatment of mRCC^[8,9]^. CARMENA demonstrated that sunitinib alone was noninferior to nephrectomy followed by sunitinib in patients with intermediate-risk or poor-risk disease, with a median OS of 18.4 months in the sunitinib-alone group *vs*. 13.9 months in the nephrectomy-sunitinib group. Notably, a portion (38 patients, 17%) of the sunitinib-alone group underwent subsequent nephrectomy for symptom control. Meanwhile, SURTIME demonstrated improved OS in patients who underwent dCN *vs*. upfront CN (intention-to-treat OS HR of 0.57, *P* = 0.03); however, this finding can only be considered hypothesis-generating as the trial closed early. In contrast to these results, retrospective studies published around the same time suggested benefits to upfront CN^[10,11]^. Bhindi *et al*.’s comparative analyses conclude that receiving multimodal therapy is associated with OS benefit, supporting upfront CN^[10]^.

## NEOADJUVANT/PREOPERATIVE

Neoadjuvant therapy offers tumor downstaging, improved resection potential, enhanced immunogenicity, and metastatic burden reduction, while enabling assessment of therapeutic efficacy^[12,13]^. Risks include overtreatment, disease progression, and adverse events (AEs), particularly immune-mediated AEs requiring high-dose steroid treatment, possibly delaying surgery. Several studies have evaluated neoadjuvant TKI use^[14–28]^, though tend to feature small sample sizes and limited focus on mRCC [Table 1].

In mRCC patients without prior nephrectomy, retrospective data demonstrated that TKI/ICI combination therapy improved OS *vs*. monotherapy, with primary tumor partial response (PR) significantly associated with better outcomes (OS and PFS)^[29]^. While this study did not evaluate CN, the 50% PR rate for dual therapy warrants investigation of neoadjuvant TKI/ICI with dCN. Studies demonstrate 10%−50% CR and 40%−50% PR in metastatic sites using ICI, with 8%−30% possible pCR at primary sites after dCN for those with complete response (CR) at metastatic sites^[30–32]^.

Initial neoadjuvant therapy safety studies in dCN demonstrated mixed results. The CheckMate 214 trial of nivolumab and ipilimumab *vs*. sunitinib in patients with advanced RCC without prior nephrectomy demonstrated better OS, overall response rate, and duration of response for the combination therapy. Importantly, a post-hoc analysis showed that every patient with CR in the trial supplement data had undergone CN^[33,34]^. A more recent evaluation of upfront ICI followed by CN demonstrated that all but one mRCC patient in a 75-patient series had viable tumor in the CN specimen after ICI^[35]^. Pignot *et al.* evaluated mRCC patients receiving neoadjuvant ICI, including ipilimumab-nivolumab and ICI/TKI for a median duration of 10 months before dCN, reporting a 63.3% rate of increased surgical difficulty, with good outcomes for responders to initial neoadjuvant therapy^[31]^. Another series of 21 mRCC patients treated with dCN after neoadjuvant ICI therapy for a median of 13 months noted negative effects on surgical planes in 38% of cases^[36]^. Questions remain regarding optimal timing, mono- *vs*. dual-therapy’s impact on surgical complexity, and systemic therapy’s contribution to perioperative morbidity.

## PERIOPERATIVE

Perioperative staging faces similar challenges to those in the neoadjuvant space. Presurgical therapy is based on clinical staging and biopsy rather than the final surgical pathology, which is limiting, as RCC tumors demonstrate heterogeneity^[37]^. ICI-based combination therapy success in mRCC has driven perioperative evaluations for improved OFS and PFS. The approach offers several advantages: higher *in situ* tumor burden may enhance immune priming and micrometastatic disease treatment^[38]^, supported by NeoAvAx data showing an increase in CD8+ densities in patients without disease recurrence^[39]^. The ADAPTeR trial showed that treatment-naive mRCC patients who responded to nivolumab had higher pre-treatment numbers of expanded T cell receptors, maintained post-treatment, suggesting the importance of pre-existing immunity in anti-PD-1 therapies^[40]^. Radiographically evaluable tumor enables treatment response assessment, guiding treatment decisions^[41]^. This may prevent missed adjuvant therapy due to surgical complications and allows histopathological examination to help guide adjuvant treatment. While offering neoadjuvant and adjuvant benefits, increased exposure increases the risk of AEs [Table 2].

The PROSPER RCC trial evaluated perioperative nivolumab in high-risk RCC patients, defined as “untreated clinical stage T2 or higher renal cell carcinoma or any clinical T stage and node-positive renal cell carcinoma of any histology planned for radical or partial nephrectomy, and no evidence of metastatic disease by CT or MRI”^[42]^. The phase III study randomized patients to perioperative nivolumab or observation with nephrectomy, but closed early when recurrence-free survival (RFS) endpoints were not met. Limitations included clinical rather than pathological staging, non-ccRCC histologies inclusion, and possible insufficient preoperative immunotherapy exposure.

Ongoing perioperative trials are crucial given the lack of prospective data regarding ICI-based therapy and patient selection. The Cyto-KIK trial (NCT04322955) is examining cabozantinib-nivolumab with 3-month neoadjuvant dosing before CN, and additional adjuvant treatment based on response^[43]^. This design addresses PROSPER RCC limitations through ccRCC focus and defined treatment duration. SWOG S1931 (PROBE, NCT04510597) is a phase III randomized trial evaluating CN’s impact on OS in ICI therapy, using ICI doublet regimens for 10–14 weeks before CN randomization^[44]^. The NORDIC-SUN trial (NCT03977571) is evaluating dCN in treatment-naive synchronous mRCC patients^[45]^, using ICI/ICI or ICI/ TKI doublets and including potentially predictive or prognostic biomarker evaluation (ctDNA, bacterial 16s ribosomal RNA, and T cell receptor genes). These studies could establish evidence-based frameworks for perioperative therapy in selected mRCC patients.

## ADJUVANT/POSTOPERATIVE

Key trials drive understanding of adjuvant therapy in mRCC. KEYNOTE-564, a phase 3 RCT, compared adjuvant pembrolizumab *vs*. placebo in high-risk ccRCC post-nephrectomy, defining high-risk disease as tumor stage 2 with grade 4 or sarcomatoid features, stage 3 or higher disease, regional lymph node metastases, or M1 NED^[46]^. FDA-approved based on disease-free survival (DFS) improvement, pembrolizumab later showed improved OS as well^[47]^. The M1 NED subgroup (58 patients) showed a HR of 0.29 for recurrence/death, though the sample size limited interpretation. Other trials showed mixed results: IMmotion010 showed no benefit with adjuvant atezolizumab compared to placebo in high-risk patients, including M1 NED patients^[48]^. EVEREST and RESORT trials with everolimus and sorafenib also failed to improve RFS^[49,50]^. The ongoing LITESPARK-022 trial (NCT05239728) is evaluating adjuvant pembrolizumab/belzutifan in intermediate-high-risk, high-risk, and M1 NED RCC^[51]^. In the context of adjuvant therapy for mRCC, M1 NED patients are those who have undergone surgical resection of the primary tumor and all resectable oligometastatic sites (synchronous or metachronous) prior to the initiation of any systemic therapy. This is distinct from those who are undergoing CN in synchronous mRCC, which includes only the resection of the primary tumor in the presence of metastatic disease. Given data demonstrating significantly improved OS using adjuvant pembrolizumab, the European Association of Urology’s recently updated guidelines strongly recommend pembrolizumab after surgery in patients with intermediate-to-high-risk ccRCC^[52]^ [Table 3].

A National Cancer Database retrospective analysis by Singla *et al*. compared OS in mRCC patients receiving modern immunotherapy (IO) with CN *vs*. IO alone, demonstrating longer median OS with IO + CN (HR 0.23 [95%CI: 0.15–0.37])^[53]^. Among 221 IO + CN patients, 197 received upfront CN, and 24 received IO before CN. The IO-first group had a lower pT stage and doubled pathologic downstaging. Multivariable analysis found CN as the only independent OS predictor, noting that only good surgical candidates (Charleson-Deyo comorbidity score ≤ 2) were included.

Dason *et al.* analyzed the National Surgical Quality Improvement Program (NSQIP) database regarding perioperative systemic therapy’s impact on CN outcomes^[54]^. Their 752-patient cohort (586 upfront CN *vs*. 166 dCN) showed no significant differences in complications. Multivariate analysis identified bleeding diathesis, adjunctive procedures, and higher ASA class - as opposed to perioperative therapy - as predictors of major complications. Three cases of wound dehiscence were reported, all occurring in the upfront CN group. Steroid use, more common in the systemic therapy group, likely for immune-related AE management, did not increase surgical risk. Current data support preoperative holding for TKI but not for ICI, with one study showing increased transfusion need with neoadjuvant ICI^[55]^. While CN post-immunotherapy appears safe in selected patients, ongoing studies will refine selection criteria and timing.

## FUTURE DIRECTIONS

Recent FDA Oncologic Drugs Advisory Committee (ODAC) panel decisions impact complex therapeutic regimen evaluations. The panel voted unanimously to mandate phase contribution assessment in cancer trials, based on the analysis of the AEGEAN trial^[56,57]^. This position emphasized benefit isolation, preventing unnecessary exposure and toxicities. Donald Berry counters with factorial study design, focusing on “main effects” rather than arm-to-arm comparisons^[58]^. He suggests that incorporating Project Optimus adaptive elements could address ODAC’s concerns without requiring prohibitively high-enrollment studies. Current phase III clinical trials for mRCC (NORDIC-SUN and PROBE) were ongoing long before new ODAC requirements. NORDIC-SUN’s multi-arm analysis would likely be in line with mandates, while PROBE’s allowance of all DFA-approved combinations may be able to satisfy requirements by factorial analysis, but may not fit technical requirements under new mandates. The formation of the International Neoadjuvant Kidney Cancer Consortium (INKCC) should address ODAC concerns as well, through cooperative data pooling^[59]^.

No approved biomarkers exist for mRCC therapy guidance. The IMDC criteria remain most predictive. The upregulated PD-1/PD-L1 pathway in genitourinary cancers contributes to immune escape^[60]^. High PD-L1 expression correlates with improved ICI outcomes in UC, but PD-L1-negative patients may also benefit, limiting its predictive reliability^[60,61]^. PD-L1 expression in RCC shows mixed utility across trials (CheckMate-214, CheckMate-025, CheckMate 9ER, CLEAR, Keynote-426). Transcriptomics clusters and liquid biopsies (ctDNA/cfDNA) may bypass PD-L1 limitations. Other biomarkers have been evaluated in the PREINSUT trial (VEGF-A, sVEGFR1, and SDF-1, among others), Javelin Renal 101 (MAdCAM-1), ADAPTeR trial (scRNA-seq for CD8+ T cell expansion), and CheckMate-214 (KIM-1), many of which correlate with outcomes in ICI and TKI settings. Spatial analysis techniques of tertiary lymphoid structures have also been associated with improved ICI response^[62,63]^. Current challenges include tumor heterogeneity, technical testing limitations, and the need for large enrollment prospective validation.

## CONCLUSIONS

Given the expansion into triplet therapy investigation as a first-line option, questions regarding the ideal selection of candidates for such therapy using predictive classification become more salient. Additional nonsurgical trials using therapies (including doublet and triplet therapies) not discussed in this article should be additionally evaluated in a perioperative context. The use of more therapies in this space may introduce improvement in OS and PFS, but will certainly be accompanied by increased AEs and side effects. Multiple avenues of active research are leading in varying directions and anticipate the addition of important data to questions surrounding the optimal stratification, treatment regimen, and timing of therapies regarding mRCC. Of importance in future trials will be novel approaches to recruitment and data analysis given new FDA ODAC requirements, investigation into novel risk stratification methods including possible advances in biomarkers, and continued investigation of systemic therapies to determine optimal timing and regimen selection.

## Figures and Tables

**Table 1.** Neoadjuvant therapy

Author, year (trial name)	Method	Treatment	Number	Population	Period of discontinuation before surgery	Mean tumor size (range) before neoadjuvant therapy	Reduction of primary tumor, median (range)	Partial response by RECIST criteria

van der Veldt *et al*., 2008^[27]^	Retrospective	Sunitinib (50 mg daily) for 4 weeks with 2-week rest between cycles	17	Advanced RCC without prior nephrectomy; clear cell and non-clear cell	NA	NA	31%	21%
Bex *et al*., 2009^[14]^	Retrospective	Sunitinib (50 mg daily) for 4 weeks with 2-week rest between cycles; dose reduction to 37.5 and 25 mg for advanced events	10	mRCC with surgically complex disease in which surgery was not feasible	NR	11.0 cm (8–15)	NA	20%
Thomas *et al*., 2009^[26]^	Retrospective	Sunitinib (50 mg daily) for 4 weeks with 2-week rest between cycles	19	Locally advanced and metastatic RCC; clear cell and non-clear cell	NA	10.5 cm (3–20)	24% (2%−46%)	11%
Hellenthal *et al*., 2010^[18]^	Prospective – single-arm, open-label	Sunitinib (37.5 mg) daily for 90 days prior to surgery	20	Biopsy-proven clear cell RCC, CN candidate, regardless of N or M stage	1–5 days prior to surgery	6.9 cm (4.4–11)	11.8% (−27% to 11%)	15%
Silberstein *et al*., 2010^[25]^	Retrospective and prospective	Sunitinib (50 mg) daily for 4 weeks with 2-week rest, for 2 cycles	12	Biopsy-proven clear cell RCC planned for partial nephrectomy, regardless of M stage	2 weeks	7.1 cm (2.6–14.5)	21.1% (3.2–45)	29%
Cowey *et al*., 2010^[15]^	Prospective - nonrandomized, open-label	Sorafenib (400 mg twice daily) for 4 weeks; dose reduction to 200 mg for grade 3 toxicities	30	cT2 or greater RCC, radiographically suspicious or biopsy-proven RCC	24–48 h	8.7 cm (4.2–14.3)	9.6% (−40% to 16%)	6%
Rini *et al*., 2012^[23]^	Phase II - single-arm	Sunitinib (50 mg) daily for 6 weeks; For patients with metastatic disease, sunitinib continued after surgery	30	Histologically or ctyologically proven RCC with unresectable primary tumor, regardless of stage; clear cell and non-clear cell	7 days	7.2 cm (1.7–20.6)	22% (−100% to 13%)	37%
Karam *et al*., 2014^[19]^	Phase II - nonrandomized, open-label	Axitinib (5 mg) twice daily for up to 12 weeks	24	Locally advanced nonmetastatic (biopsy-proven) clear cell RCC	36 h	10.0 cm (4.2–16.6)	28.3% (5.3–42.9)	45.8%
Rini *et al*., 2015^[24]^	Phase II - single-arm	Pazopanib (800 mg) daily for up to 16 weeks; dose reduction to 400 or 600 mg for unacceptable toxicity	25	Localized, biopsy-proven clear cell RCC	7 days	7.3 cm (2.3–10.7)	26% (−44% to 1%)	36%
Zhang *et al*., 2015^[28]^	Retrospective	Sorafenib (400 mg) twice daily; dose reduction to 600 mg daily	18	RCC of any stage who received sorafenib preoperatively	7–30 days	7.8 cm (3.6–19.2)	NA	22%
Lane *et al*., 2015^[21]^	Retrospective - multi-institutional	Sunitinib (50 mg) daily for 4 weeks with 2-week rest for 2 cycles, or (37.5 mg) daily for 90 days	72	RCC, candidate for partial nephrectomy	5–14 days	7.2 cm (5.3–8.7)	32% (14–46)	19%
Hatiboglu *et al*., 2017^[17]^	Prospective - singlecenter, randomized, placebo-controlled, double-blind, pilot	Sorafenib (400 mg) twice daily for 28 days vs. placebo	12	cT1-T3 RCC, N0, M0, candidate for curative surgery	12 h	5.4 cm (4.3–7.3)	29% (−4.9% to 61%)	NR
Lebacle *et al*., 2019 (AXIPAN)^[22]^	Phase II - multicenter, open-label, non-randomized	Axitinib (5 mg) twice daily, uptitrated (up to 10 mg twice daily) based on tolerance for 2–12 months	18	CT2aN0NxM0 clear cell RCC	3–10 days	7.6 cm (7.0–9.8)	17.1% (4.8–29.4)	22%
de Velasco *et al*., 2020 (CABOPRE)^[16]^	Phase II - multicentered, open-label ongoing	Cabozantinib (60 mg) daily for 12 weeks	18 (target)	Locally advanced or metastatic RCC	NR	NR	NR	27%
Meerveld-Eggink *et al*., 2022^[30]^	Retrospective	Nivolumab + Ipilumab	71	Treatment-naive mRCC	NR	9.3 cm (2.5–16.1)	12.9% (−75% to 29%) for intermediate-risk IMDC; 17.6% (−67% to 29%) for high-risk IMDC	33%
Pignot *et al*., 2022^[31]^	Retrospective	Nivolumab + ipilumab, ICI +TKI, or nivolumab alone, 3–38 months	31	Metastatic RCC, complete or partial response neoadjuvant ICI-based therapy prior to surgery	NR	NR	NR	NR
Shirotake *et al*., 2022^[32]^	Retrospective	Nivolumab + Ipilimumab; 27–216 days (for those who underwent delayed CN)	79 (10 patients underwent CN)	Advanced RCC, IMDC intermediate- and poor-risk; clear and non-clear cell histology	NR	NR	NR	50.8%
Graafland *et al*., 2022^[36]^	Retrospective	Nivolumab + Ipilimumab, or	21	mRCC with partial or complete response prior to CN	0–22 months	NR	NR	NR
Bickley *et al*., 2024^[29]^	Retrospective	TKI only for a mean of 3.2 months (IQR 3.7) until best tumor diameter change	28	Treatment-naive mRCC	NR	NR	−8.0%	14%
		ICI only for a mean of 3.0 months (IQR 2.9) until best tumor diameter change	23 (2 underwent delayed CN)		NR	NR	+5.1 %	22%
		ICI + TKI for a mean of 3.9 months (IQR 5.) until best tumor diameter change	14 (2 underwent delayed CN)		NR	NR	−31.1 %	50%

**Table 2.** Clinical trials for perioperative therapy

Trial	Method	Treatment	Number	Population	Primary outcome	Results

Allaf *et al*., 2024 (PROSPER RCC)^[42]^	Phase III - open-label	Nivolumab (480 mg) prior to CN with nine adjuvant doses *vs*. CN alone	819	Treatment-naive cT2 or greater, or N+ RCC, M1 NED; clear cell and non-clear cell histology	RFS	No significant difference. (125 [33%] of 381 had recurrence-free survival events) *vs*. surgery only (133 [33%] of 399; hazard ratio 0.94 [95%CI: 0.74–1.21]; one-sided *P* = 0.32)
Runcie *et al*., 2021 (Cyto-KIK)[43]	Phase II - open-label	Cabozantinib (40 mg) daily and nivolumab (480 mg) every 4 weeks for 12 weeks prior to CN, with resumption of treatment postoperatively	48 (target)	Treatment-naive mRCC; clear cell only	CR	Ongoing study
Vaishampayan *et al*., 2022 (PROBE - SWOG S1931)[44]	Phase III	ICI-based combination treatment* followed by randomization at 10–14 weeks to either: CN *vs* continuation of ICI-based combination treatment**	364 (target)	Previously treated and treatment naïve mRCC; clear cell and nonclear cell histology	OS	Ongoing study
Iisager *et al*., 2024 (NORDIC-SUN)^[45]^	Phase III - open-label	3 months of ICI-based systemic therapy followed by deferred CN *vs*. 3 months of ICI-based therapy followed by maintenance nivolumab or a TKI-IO-combination	400 (target)	Treatment-naive mRCC; clear cell and non-clear cell histology	OS	Ongoing study

* Either of three regimens: (1) Nivolumab + ipilimumab, (2) Pembrolizumab + axitinib, (3) Avelumab + axitinib;

** After randomization, those in the systemic therapy alone group received nivolumab, pembrolizumab, avelumab, and axitinib

**Table 3.** Clinical trials for adjuvant therapy

Trial	Method	Treatment	Treatment duration	Number	Population	Primary outcome	Results

Haas *et al*., 2008	Phase III - (ASSURE)^[64]^ double-blind	Sorafenib or sunitinib *vs*. placebo	54 weeks	1,943	High-grade clear cell or non-clear RCC within 12 weeks of CN	DFS	No significant difference. Sunitinib: 5.8 years HR, 1.02; 97.5%CI: 0.85 to 1.23; *P* = 0.8038 Sorafenib: 6.1 years HR, 0.97; 97.5%CI: 0.80 to 1.17; *P* = 0.7184 Placebo: 6.6 years
Ravaud *et al*., 2016 (S- TRAC)[65]	Phase III - double-blind	Sunitinib *vs*. placebo	1 year	615	Locoregional clear cell RCC only (T3 or higher, regional lymph node metastasis, or both)	DFS	Significant difference. Sunitinib: 6.8 years HR, 0.76; 95%CI: 0.59 to 0.98; *P* = 0.03 Placebo: 5.6 years
Motzer *et al*., 2021 (PROTECT)^[66]^	Phase III - double-blind	Pazopanib *vs*. placebo	1 year	1,538	High-grade nonmetastatic clear cell RCC or predominant clear cell histology	DFS	No significant difference (HR, 0.86; 95%CI: 0.70 to 1.06; *P* = 0.165)
Gross-Goupil *et al*., 2018 (ATLAS)^[67]^	Phase III - double-blind	Axitinib *vs*. placebo	1–3 years	724	High-grade nonmetastatic clear cell predominant RCC	DFS	No significant difference (HR, 0.870; 95%CI: 0.660 to 1.147; *P* = 0.3211)
Eisen *et al*., 2020 (SORCE)[68]	Phase III - double-blind, three-arm	Sorafenib (3 years) *vs*. Sorafenib (1 year) *vs*. placebo	1 or 3 years	1,711	Intermediate or high-risk RCC, clear cell and non-clear cell histology	DFS	No significant difference (HR, 1.01; 95%CI: 0.83 to 1.23; *P* = 0.95)
Ryan *et al*., 2021 (EVEREST)^[49]^	Phase III - double blind	Everolimus *vs*. placebo	Nine cycles of 6 weeks	1,545	Intermediate or high-risk RCC, clear cell and non-clear cell histology	DFS	No significant difference at interim analysis (HR, 0.85; 95%CI: 0.72–1.00; *P* = 0.0246)
Choueiri *et al*., 2021 (Keynote 564)^[46]^	Phase III - double-blind	Pembrolizumab *vs*. placebo	1 year	994	Locoregional clear cell RCC or metastatic disease with high risk of recurrence	DFS	Significant difference - improved DFS in treatment group *vs*. placebo (HR 0.63, 95%CI: 0.50–0.80)
Pal *et al*., 2022 (IMmotion10)^[48]^	Phase III - double-blind	Atezolizumab (1,200 mg) *vs*. placebo	16 cycles or 1 year	778	RCC with clear cell or sarcomatoid component and increased risk of recurrence	DFS	No significant difference (HR 0.93, 95%CI: 0.75–1.115, *P* = 0.50)
Motzer *et al*., 2023 (Checkmate 914)^[69]^	Phase III – open-label, double-blind	Nivolumab + ipilumab *vs*. placebo	24–36 weeks	1,628	Localized clear cell RCC at high risk of relapse after radical or partial nephrectomy between 4–12 weeks before randomization	DFS	DFS was not met and was not statistically significant between groups (HR 0.92, 95%CI: 0.71–1.19; *P* = 0.53)
Oza *et al*., 2021 (RAMPART)^[70]^	Phase III – multi-arm, multi-stage	Active surveillance *vs*. Durvalumab alone *vs*. Druvalumab + Trelelimumab	1 year	1,750 (target)	Intermediate and high-risk Leibovich score	DFS and OS	Ongoing study
